# Co-overexpression of AXL and c-ABL predicts a poor prognosis in esophageal adenocarcinoma and promotes cancer cell survival

**DOI:** 10.7150/jca.47318

**Published:** 2020-08-08

**Authors:** Jun Hong, Fatma Abid, Sharon Phillips, Safia N. Salaria, Frank L. Revetta, Dunfa Peng, Mary K. Washington, Wael El-Rifai, Abbes Belkhiri

**Affiliations:** 1 Department of Surgery, Vanderbilt University Medical Center, Nashville, TN, USA.; 2 Vanderbilt Center for Quantitative Sciences, Department of Biostatistics, Vanderbilt University Medical Center, Nashville, TN, USA.; 3 Department of Pathology, Microbiology and Immunology, Vanderbilt University Medical Center, Nashville, TN, USA.; 4 Department of Surgery, University of Miami Miller School of Medicine, Miami, FL, USA.; 5 Vanderbilt-Ingram Cancer Center, Vanderbilt University Medical Center, Nashville, TN, USA.

**Keywords:** Esophageal adenocarcinoma, Prognosis, Cell survival, AXL, c-ABL, Necrosis

## Abstract

**Background:** Esophageal adenocarcinoma (EAC) is highly aggressive and characterized by poor prognosis. AXL expression has been linked to Barrett's tumorigenesis and resistance to chemotherapy, which is associated with c-ABL intracellular localization. However, the molecular and functional relationship between AXL and c-ABL and the clinical significance of the co-expression of these proteins in EAC remain unclear.

**Methods:** We used immunohistochemical analysis (IHC) on tissue microarrays containing human EAC samples (n=53) and normal esophageal tissues (n=11) in combination with corresponding deidentified clinicopathological information to evaluate the expression and the prognostic significance of AXL and c-ABL in EAC. The data were statistically analyzed using Kruskal-Wallis, the chi-square, the Fisher's exact, and Pearson tests. The Kaplan-Meier method and Cox proportional hazards regression model were used to evaluate cancer patient survival. We used a serum deprivation EAC cell model to investigate the pro-survival function of AXL and c-ABL using cell viability, apoptosis, and lactate dehydrogenase activity assays. We performed *in vitro* assays, including Western blotting, quantitative real-time PCR, and translational chromatin immunoprecipitation (TrIP-Chip) to study the molecular relationship between AXL and c-ABL in EAC cells.

**Results:** IHC analysis revealed that AXL and c-ABL were overexpressed in 55% and 66% of EAC samples, respectively, as compared to normal tissues. Co-overexpression of the two proteins was observed in 49% of EAC samples. The chi-square test indicated a significant association between AXL and c-ABL expression in the EAC samples (χ^2^ = 6.873, *p =* 0.032), and the expression of these proteins was significantly associated with EAC patient age (*p <* 0.001), tumor stage (*p <* 0.01), and lymph node status (*p <* 0.001). AXL and c-ABL protein expression data analysis exhibited an identical clinicopathological association profile. Additionally, we found a significant association between expression of AXL (χ^2^ = 16.7, *p =* 0.002) or c-ABL (χ^2^ = 13.4, *p =* 0.001) and survival of EAC patients. The Cox proportional hazards model and log rank test predicted a significant increase in mortality of patients with high expression of AXL [hazard ratio (HR): 2.86, 95% confidence interval (CI): 1.53 - 5.34, *p =* 0.003] or c-ABL [HR: 3.29, 95% CI: 1.35 - 8.03, *p =* 0.001] as compared to those patients with low expression of AXL or c-ABL proteins. Molecular investigations indicated that AXL positively regulates c-ABL protein expression through increased cap-dependent protein translation involving phosphorylation of EIF4E in EAC cells. Next, we investigated the functional relationship between AXL and c-ABL in EAC cells. We demonstrated that the pro-survival activity of AXL requires c-ABL expression in response to serum deprivation.

**Conclusion:** This study highlights the importance of the co-overexpression of AXL and c-ABL proteins as a valuable prognostic biomarker and targeting these proteins could be an effective therapeutic approach in EAC or other solid tumors expressing high levels of AXL and c-ABL proteins.

## Introduction

The incidence of esophageal adenocarcinoma (EAC), which is a major histologic subtype of esophageal cancer, has rapidly increased in many Western countries in the past few decades 1, 2. Gastroesophageal reflux disease and associated premalignant metaplasia of the esophagus (Barrett's esophagus) have been identified as major risk factors for EAC 3, 4. Because of the late presentation of symptoms and the aggressive nature of EAC, the overall prognosis remains poor. In fact, the 5-year survival rates for patients with stage III and IV tumors are 17.6% and 2.1%, respectively (reviewed in 5). The increasing overexpression of AXL receptor tyrosine kinase, which is a member of the TAM family that also includes TYRO 3 and MER, has been associated with Barrett's tumorigenesis and poor prognosis in EAC 6. We have previously reported that AXL promotes resistance to tumor necrosis factor-related apoptosis-inducing ligand (TRAIL) 7, cisplatin 8, and epirubicin 9 in EAC cells.

The human c-ABL protein, which is encoded by *ABL1* gene, is a non-receptor tyrosine kinase that regulates cell migration, proliferation, responses to DNA damage and oxidative stress, and cell survival 10, 11. The oncogenic function of c-ABL as a constitutively active fusion protein (BCR-ABL) has been initially well established in hematopoietic cancers 12. Additionally, increased protein expression and activation of c-ABL have been associated with progression of solid tumors 13, 14. Inhibition of c-ABL kinase activity suppresses pro-tumorigenic functions, such as cell proliferation, anchorage-independent growth, and survival in response to nutrient deprivation in breast cancer cells 15. Moreover, c-ABL has been shown to promote the expression and activity of EGFR by preventing dephosphorylation or internalization of the membrane EGFR receptor 16, 17. Notably, targeting c-ABL with the clinically approved inhibitor imatinib (STI571) can sensitize resistant breast cancer cells to lapatinib, a dual tyrosine kinase inhibitor specific for HER2 and EGFR 18. In a published report, we showed that AXL expression promotes cisplatin resistance by preventing c-ABL from translocating to the nucleus in response to DNA damage in EAC cells 8. However, the functional and molecular signaling relationship between AXL and c-ABL, and the clinical significance of their expression in esophageal adenocarcinoma remain not fully investigated.

In the current study, we have shown a novel mechanism by which AXL promotes c-ABL protein expression through up-regulation of protein translation and demonstrated that c-ABL mediates the pro-survival function of AXL in EAC cells. Additionally, we have established that frequent overexpression of AXL and c-ABL proteins predicts poor prognosis for EAC patients.

## Materials and Methods

### Cell culture and reagents

The human EAC cancer cell line, OE33, was purchased from Sigma-Aldrich (St. Louis, MO, USA). FLO-1 cells were kindly provided by D. Beer (University of Michigan, Ann Arbor, MI, USA). FLO-1 cells were cultured in Dulbecco's modified Eagle's medium (DMEM; GIBCO, Carlsbad, CA, USA) supplemented with 5% fetal bovine serum (FBS, Invitrogen Life technologies, Carlsbad, CA, USA) and 1% penicillin/streptomycin (GIBCO). OE33 cells were cultured in RPMI medium (GIBCO) supplemented with 5% FBS and 1% penicillin/streptomycin. All cell lines were authenticated using short tandem repeat (STR) profiling (Genetica DNA Laboratories, Burlington, NC, USA). AXL, c-ABL, and β-actin antibodies were purchased from Cell Signaling Technology (Danvers, MA, USA). Flag antibody was obtained from Sigma-Aldrich. Mouse and rabbit secondary antibodies were purchased from Promega Corporation (Madison, WI, USA).

### AXL and c-ABL expression and knockdown

The pcDNA4/AXL-Myc-His and pcDNA4 plasmids, kindly provided by R.M. Melillo (University of Naples, Italy), were used to generate stable cell lines. OE33 cells were stably transfected using lipofectamine 2000 (Invitrogen) and selected with 100 µg/ml zeocin (Invitrogen) following standard protocols 19. For stable knockdown of AXL expression in FLO-1 cells, a control shRNA or a pool of five validated AXL shRNA lentivirus particles (Sigma-Aldrich) were used. Cells were selected with 1 µg/ml puromycin for 10 days. The stable expression of c-ABL in FLO-1/shAXL cells was achieved by transfection of pcMV3/Flag-ABL1 (Sino Biological) using lipofectamine 2000, followed by selection with 100 µg/ml hygromycin (Invitrogen) for 2 weeks. For stable knockdown of c-ABL expression, control shRNA or a pool of five validated ABL1 shRNA lentivirus particles (Sigma-Aldrich) were used to transduce OE33/AXL cells, followed by selection with 1 µg/ml puromycin for 10 days.

### Quantitative real-time PCR analysis

Total RNA was isolated from cells using Trizol (Invitrogen). A Sensi-FAST cDNA Synthesis Kit (Bioline, Taunton, MA, USA) was used to synthesize cDNA. The quantitative real-time reverse transcriptase PCR (qRT-PCR) was performed with a Bio-Rad CFX connect Real-time System in 10-µl reaction volume using iQ SYBR Green Supermix (Bio-Rad Laboratories Inc., Hercules, CA, USA). The threshold cycle number was calculated by Bio-Rad CFX manager software version 3.0. The qRT-PCR reactions were performed in triplicate following standard protocols 9, 20. All primers were purchased from Integrated DNA Technologies (Supplemental Table S3).

### Isolation of polysome-associated mRNA transcripts

Translational chromatin immunoprecipitation analysis (TrIP-Chip assay) was used to isolate polysome-associated mRNA transcripts as described previously 21. Briefly, cells from 10-cm culture dishes were harvested with 0.05% Trypsin-EDTA (Invitrogen). Cells (10^7^) were then incubated with 800 µl DMEM, supplemented with 5% FBS and 100 μg/ml cycloheximide (CHX; Sigma-Aldrich), at 37°C for 5 min. Next, 200 μl of 1 mM DSP (succinimidyl propionate; Pierce, IL, USA) were added as a cross-linking reagent followed by incubation for 5 min at 37°C. The cells were then harvested by centrifugation and washed twice with ice-cold PBS containing 100 μg/ml CHX. The pellets were incubated for 20 min in 500 μl of low-salt buffer (LSB) (20 mM HEPES, pH 7.4, 100 mM KCl, 2 mM MgCl2) containing 1 mM dithiothreitol and then lysed in 200 μl LSB containing 1.2% Triton X-100. After centrifugation, 350 μl of each lysate were incubated with HSP70/HSP73 antibody-conjugated magnetic beads for 2 h at 4°C. The polysome complexes containing translationally active mRNA transcripts were extracted using the Trizol reagent according to the manufacturer's instructions (Invitrogen). cDNA was synthesized with the SensiFAST™ cDNA Synthesis Kit (Swedesboro, NJ, USA), and subjected to qRT-PCR analysis.

### Western blotting

Cells were pelleted and lysed with RIPA buffer supplemented with protease and phosphatase inhibitors (Santa Cruz Biotechnology). Western blotting analysis was performed using laboratory standard methods as described previously 22.

### Apoptosis assay

Cells (10^5^ cells per well) were seeded in 6-well-plates. The following day, cells were cultured in 5% FBS or 0% FBS media for 24 h or 48 h. Cells were then collected and stained with Annexin-V fluorescein isothiocyanate (FITC) and propidium iodide (PI; R&D Systems). The cells were next subjected to fluorescence-activated cell sorting (FACS) analysis by a flow cytometer (Becton Dickinson, Franklin Lakes, NJ, USA).

### Cell viability assay

Cells were seeded in 6-well plates at 2x10^5^ cells per well and cultured in 5% FBS overnight. Viable cells (control) in one plate for each cell line were then counted following Trypan blue staining. The medium was replaced with fresh medium containing 0% FBS in the remaining plates and the cell lines were cultured for 2, 4, and 6 days, and cell viability was measured by counting cells after Trypan blue staining. The data were normalized to viable control cells and presented as relative cell survival.

### Clonogenic cell survival assay

Cells were seeded in 6-well plates at 5000 cells per well and cultured in 5% FBS overnight. Next, the cells were cultured without serum for 24 h. The medium was then replaced with fresh medium containing 5% FBS and the cells were grown for 1 week to 10 days to allow for formation of colonies. The colonies were stained and assessed following standard protocols 9.

### Lactate dehydrogenase activity assay

Cells were seeded in 96-well plates at 5x10^3^ cells per well and cultured in 5% FBS overnight. The cells were then cultured in 0% or 5% FBS for 24 h. As a measure of necrotic cell death, lactate dehydrogenase activity was measured using LDH Cytotoxicity WST Assay (Enzo Life Sciences, Farmingdale, NY, USA) according to the manufacturer's instructions.

### Immunohistochemistry and clinicopathological data

Tissue microarrays (TMA) containing 64 de-identified archival human esophageal tissue samples (11 normal and 53 adenocarcinomas), and the corresponding patient clinicopathological information (Supplemental Table S2) were provided by Vanderbilt-Ingram Cancer Center's gastrointestinal Specialized Program of Research Excellence (SPORE). TMA sections were subjected to immunohistochemical (IHC) staining of AXL receptor tyrosine kinase with polyclonal goat AXL antibody (1:200; AF154; R&D Systems) or c-ABL kinase with polyclonal rabbit c-ABL antibody (1:50; NBP1-90288; Novus Biologicals) following standard protocols 9. The immunoreactivity was evaluated by two trained pathologists. The immunostaining of AXL was scored as 0, absent, 1+, weak (1-33%), 2+, moderate (34-66%), and 3+, strong (67-100%). The c-ABL IHC staining intensity was scored as 1 (weak), 2 (moderate) and 3 (strong). The frequency of neoplastic cells demonstrating IHC staining was graded as the percentage of neoplastic cells in the core showing positive labeling: 0, absent, 1 (1-25%), 2 (26-50%), 3 (51-75%), and 4 (greater than 75%). The IHC index score was calculated by multiplying the intensity and frequency. We stratified the tumor samples into 3 groups. Low expression group (AXL, index score ≤ 1; c-ABL, index score ≤ 2), medium expression group (AXL, index score = 2; c-ABL index score = 4), and high expression group (AXL, index score = 3; c-ABL, index score ≥ 8).

### Statistical analysis

Data of the* in vitro* studies were expressed as mean ± SD of three independent experiments. Statistical analysis was conducted with the GraphPad Prism 8.3. Statistical significance between two groups was analyzed by the Student's *t* test. For the IHC study of tumor tissues and corresponding patient clinical data, all the statistical analyses were performed using the statistics program R version 3.4.4. Kruskal-Wallis test was used for comparison between continuous data. For comparison between categorical data, the χ^2^ test, Fisher's exact test, and Pearson test were used. Expression levels of AXL and c-ABL were assessed for association with gender, age, tumor size, tumor categories, tumor stage, N/T classification, tumor differentiation, and lymph nodes. Cancer patient survival was evaluated by Kaplan-Meier method and Cox proportional hazards regression model. Differences with p ≤ 0.05 were considered significant.

## Results

### Frequent co-overexpression of AXL and c-ABL proteins in esophageal adenocarcinoma

**Figure 1:**
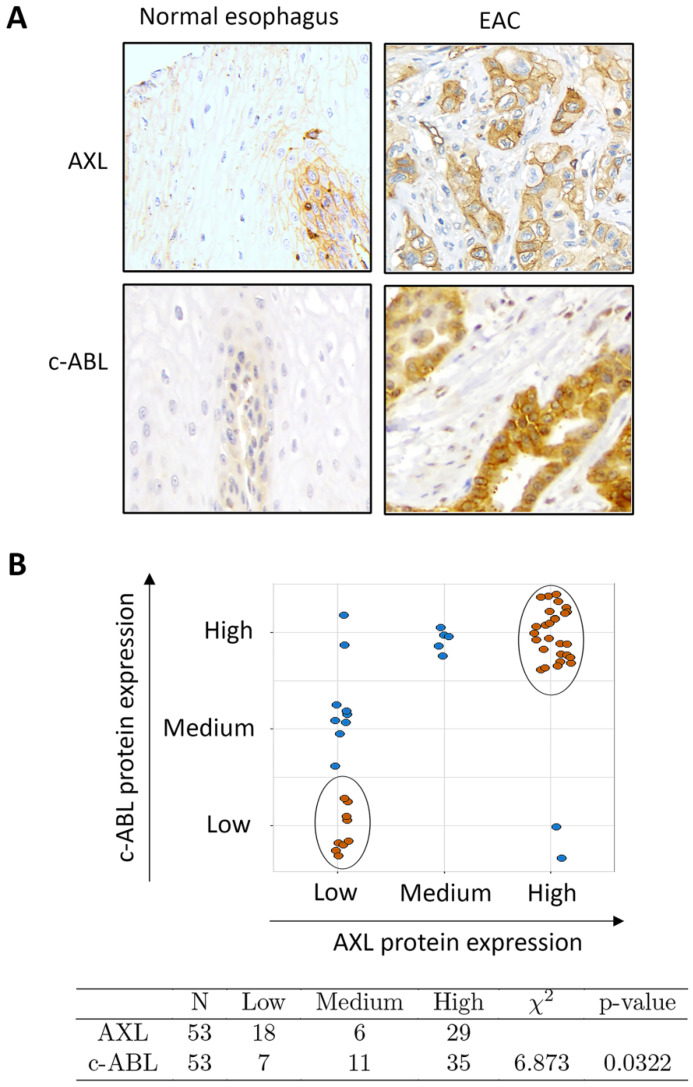
** Association between AXL and c-ABL protein expression in esophageal adenocarcinomas. A,** Representative images (200x magnification) of immunohistochemical staining (brown color) of AXL and c-ABL in human normal esophageal epithelial tissue samples (weak staining, left) and moderately differentiated esophageal adenocarcinoma tissues (strong staining, right). **B,** Tissue samples with matching expression levels of AXL and c-ABL are indicated in brown color (upper panel). The chi-square test (χ^2^) was used to assess the association between AXL and c-ABL protein expression in tumor samples (lower panel).

The results from Western blot analysis showed increased protein co-expression of AXL and c-ABL in the examined EAC cell lines (OE33, FLO-1, and SK-GT-4) relative to preneoplastic Barrett's cells (CP-A, BAR-T, and CP-B) and normal squamous esophageal cells (EPC2) (Supplemental Figure S1). We next evaluated AXL and c-ABL protein expression in tissue microarrays containing 53 EAC and 11 esophageal normal squamous tissue samples by IHC staining with anti-AXL or anti-c-ABL specific antibodies. The IHC results indicated that the protein expression of AXL and c-ABL was generally low in normal squamous tissue samples (Figure 1A) and high in EAC samples as indicated by strong cytoplasmic and membrane staining for AXL and mostly cytoplasmic staining for c-ABL (Figure 1A)**.** The analysis of the IHC data, based on the index scores cutoff of 3 (AXL) or 8 (c-ABL) to indicate overexpression, revealed that AXL and c-ABL were overexpressed in 55% and 66% of EAC tissue samples, respectively (Supplemental Table S1). The tested normal tissue samples did not exhibit overexpression of AXL or c-ABL (Supplemental Table S1). The difference in AXL (*p <* 0.001) or c-ABL (*p <* 0.001) protein expression between normal and EAC tissue samples was statistically significant (Pearson test). We next investigated whether there was a significant association between AXL and c-ABL protein expression in EAC tissue samples. Based on expression levels, we stratified the tumor samples into 3 groups (low, medium, and high) as described in Materials and methods. The data analysis revealed 26 tumor samples with matching high expression of both AXL and c-ABL out of 53 total samples. In addition, there were 9 non-matching samples with high expression of either AXL or c-ABL (Figure 1B, upper panel). The statistical analysis of the IHC data using the chi-square test showed a relatively strong association between AXL and c-ABL protein expression in the tested EAC samples (χ^2^ = 6.873, *p =* 0.032) (Figure 1B, lower panel).

### Association between AXL or c-ABL expression and clinicopathological features in EAC patients

To investigate whether AXL and c-ABL expression levels are linked to clinicopathological features in EAC, we analyzed our data that included AXL and c-ABL protein expression in tumor samples and their corresponding clinicopathological information from 53 EAC patients (Supplemental Table S2). As summarized in Table 1, the Fisher's exact test indicated that AXL expression was significantly associated with patient age (*p <* 0.001) and lymph node status (*p <* 0.001). In addition, there was a significant association between the tumor stage and AXL expression as indicated by the Pearson test (*p <* 0.001). However, the expression of AXL had no significant association with gender, tumor size, T/N classification, and differentiation (all *p >* 0.05). Similarly, as shown in Table 2, the Fisher's exact test revealed that the expression of c-ABL was significantly associated with age (*p <* 0.001) and lymph node status (*p <* 0.001). Additionally, the Pearson test indicated a significant association between c-ABL expression and tumor stage (*p <* 0.001). Conversely, there was no significant association between c-ABL expression, gender, tumor size, T/N classification, and differentiation (all *p >* 0.05). Taken together, the data clearly showed that both AXL and c-ABL have an identical clinicopathological association profile and the concurrent expression of these two proteins was significantly associated with EAC patient age, tumor stage, and lymph node status.

### Elevated expression of AXL and c-ABL is associated with poor prognosis in EAC patients

**Figure 2:**
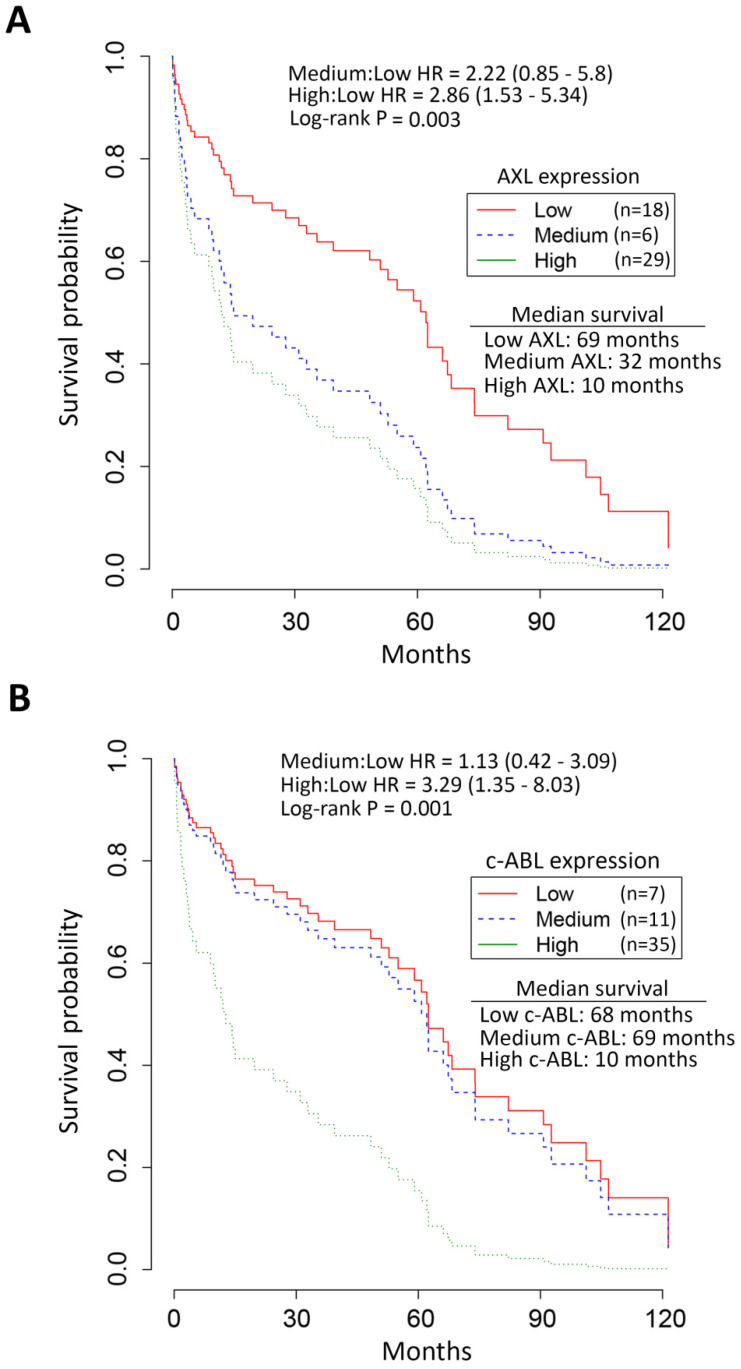
** Association of high AXL and c-ABL expression with low survival of EAC patients.** Survival analysis of AXL and c-ABL protein expression in EAC patients (n = 53) by Kaplan-Meier method, the Cox proportional hazards model, and the log-rank test. **A,** EAC patients with high (n = 29, green line) AXL expression had significantly worse overall survival than EAC patients with low (n = 18, red line) or medium (n = 6, blue line) AXL expression. The hazard ratio (HR) predicted a significant increase in mortality of EAC patients with high AXL expression relative to patients with low AXL expression. **B,** EAC patients with high (n = 35, green line) c-ABL expression had significantly worse overall survival than EAC patients with low (n = 7, red line) or medium (n = 11, blue line) c-ABL expression. HR data predicted a significantly increased mortality in EAC patients with high c-ABL expression as compared to patients with low c-ABL expression.

Survival analysis by Kaplan-Meier revealed the median overall survival of EAC patients with high expression of AXL (10 months, Figure 2A) and c-ABL (10 months, Figure 2B) as opposed to those with low expression of AXL (69 months, Figure 2A) and c-ABL (68 months, Figure 2B). The chi-square test indicated a significant association between expression of AXL (χ^2^ = 16.7, *p =* 0.002) or c-ABL (χ^2^ = 13.4, *p =* 0.001) and survival of EAC patients (Figure 2). The Cox proportional hazards model and log rank test predicted a significant increase in mortality of EAC patients with high expression of AXL [hazard ratio (HR): 2.86, 95% confidence interval (CI): 1.53 - 5.34, *p =* 0.003] or medium expression of AXL [HR: 2.22, CI: 0.85 - 5.8, *p =*0.003] relative to those patients with low expression of AXL (Figure 2A). Similarly, the analysis predicted a significant increase in mortality of EAC patients with high expression of c-ABL [HR: 3.29, CI: 1.35 - 8.03, *p =* 0.001] or medium expression of c-ABL [HR: 1.13, CI: 0.42 - 3.09, *p =* 0.001] as compared to those patients with low expression of c-ABL (Figure 2B). Together, the data clearly indicated that EAC patients with high expression of AXL and c-ABL had worse survival and poor prognosis than those with low expression.

### AXL enhances c-ABL protein expression through regulation of cap-dependent translation

**Figure 3:**
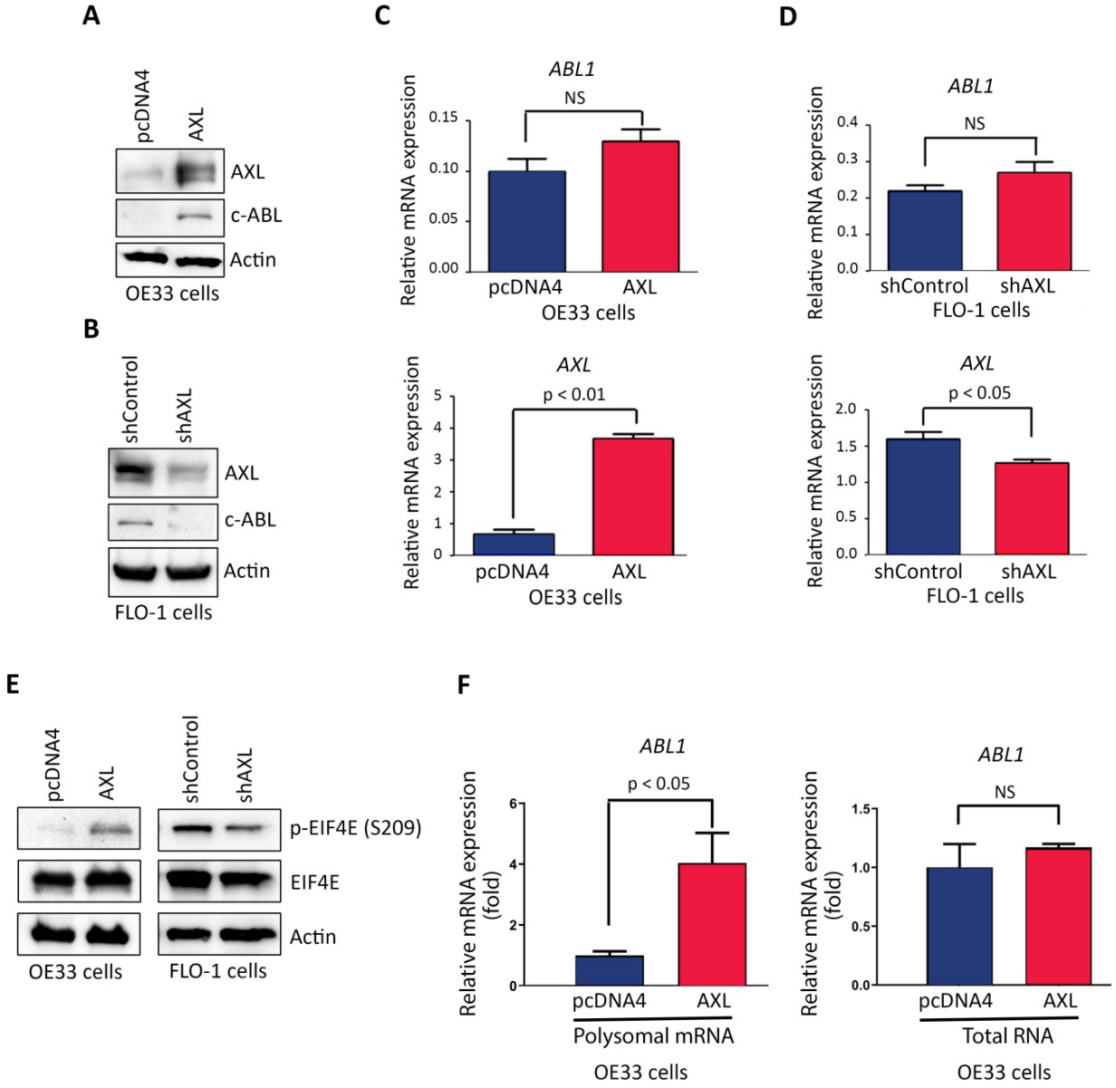
** AXL promotes c-ABL protein expression through regulation of cap-dependent translation.** Western blotting and quantitative real-time PCR (qRT-PCR) analysis of AXL and c-ABL protein and mRNA expression following overexpression of AXL in OE33 cells (**A&C**) or knockdown of AXL in FLO-1 cells (**B&D**) relative to control cells. **E,** Western blot analysis of p-EIF4E (S209) and EIF4E proteins after overexpression of AXL in OE33 cells (left panel) or knocking down of AXL expression in FLO-1 cells (right panel) as compared to control cells. **F,** qRT-PCR analysis of *ABL1* mRNA expression in polysomal mRNA (left panel) or total RNA (right panel) after overexpression of AXL in OE33 cells relative to control cells. The data are representative of three independent experiments. NS, not significant.

Based on our data showing that AXL and c-ABL are concurrently overexpressed in 26 tumors (49%) out of total 53 EAC samples, and a significant association between the expression of the two proteins (Figure 1B), we investigated whether AXL regulates c-ABL expression in EAC cells. Indeed, Western blot analysis indicated that overexpression of AXL increased c-ABL protein level in OE33 cells (Figure 3A) and knocking down of AXL expression decreased c-ABL protein expression in FLO-1 cells (Figure 3B). However, qRT-PCR analysis showed that overexpression of AXL in OE33 cells (Figure 3C) or knockdown of AXL in FLO-1 cells (Figure 3D) had no significant effects on *ABL1* mRNA expression.

To identify the molecular mechanism by which AXL regulates c-ABL expression, we investigated whether AXL expression through a posttranslational mechanism could affect c-ABL protein stability. We transiently co-transfected HEK-293 cells with ABL1 and pcDNA3 or AXL plasmids and assessed c-ABL protein levels by Western blot analysis after treatment with cycloheximide (CHX). The data indicated that AXL expression did not enhance c-ABL protein stability (Supplemental Figure S2). In fact, exogenous AXL expression appeared to reduce c-ABL protein stability in our experimental cell model. After ruling out protein stability as a molecular mechanism, we next investigated whether AXL could enhance cap-dependent translation, thereby increasing c-ABL protein expression. We postulated that AXL promotes phosphorylation of eukaryotic translation initiation factor (EIF4E), the mRNA cap-binding protein, in EAC cells. Western blot analysis data indicated that overexpression of AXL increased expression of p-EIF4E (S209) protein in OE33 cells (Figure 3E, left panel). Conversely, knocking down of endogenous AXL expression decreased p-EIF4E (S209) protein level in FLO-1 cells (Figure 3E, right panel). We next investigated if EIF4E cap-dependent translation involves mRNA translation of *ABL1* in OE33 cells. We used the translational chromatin immunoprecipitation analysis (TrIP-ChIP assay), which captures actively translated polysomal mRNAs, followed by quantitative qRT-PCR analysis. We found that overexpression of AXL in OE33 cells significantly increased the level of translated polysomal mRNA of *ABL1* relative to control cells (*p <* 0.05, Figure 3F, left panel). However, qRT-PCR analysis of total RNA indicated no significant effect on *ABL1* mRNA expression following overexpression of AXL in OE33 cells relative to control cells (Figure 3F, right panel). Together, our data strongly suggested that AXL-dependent induction of c-ABL protein expression involves increased cap-dependent protein translation through phosphorylation of EIF4E.

### AXL requires c-ABL expression to protect cancer cells from serum deprivation-induced death

**Figure 4:**
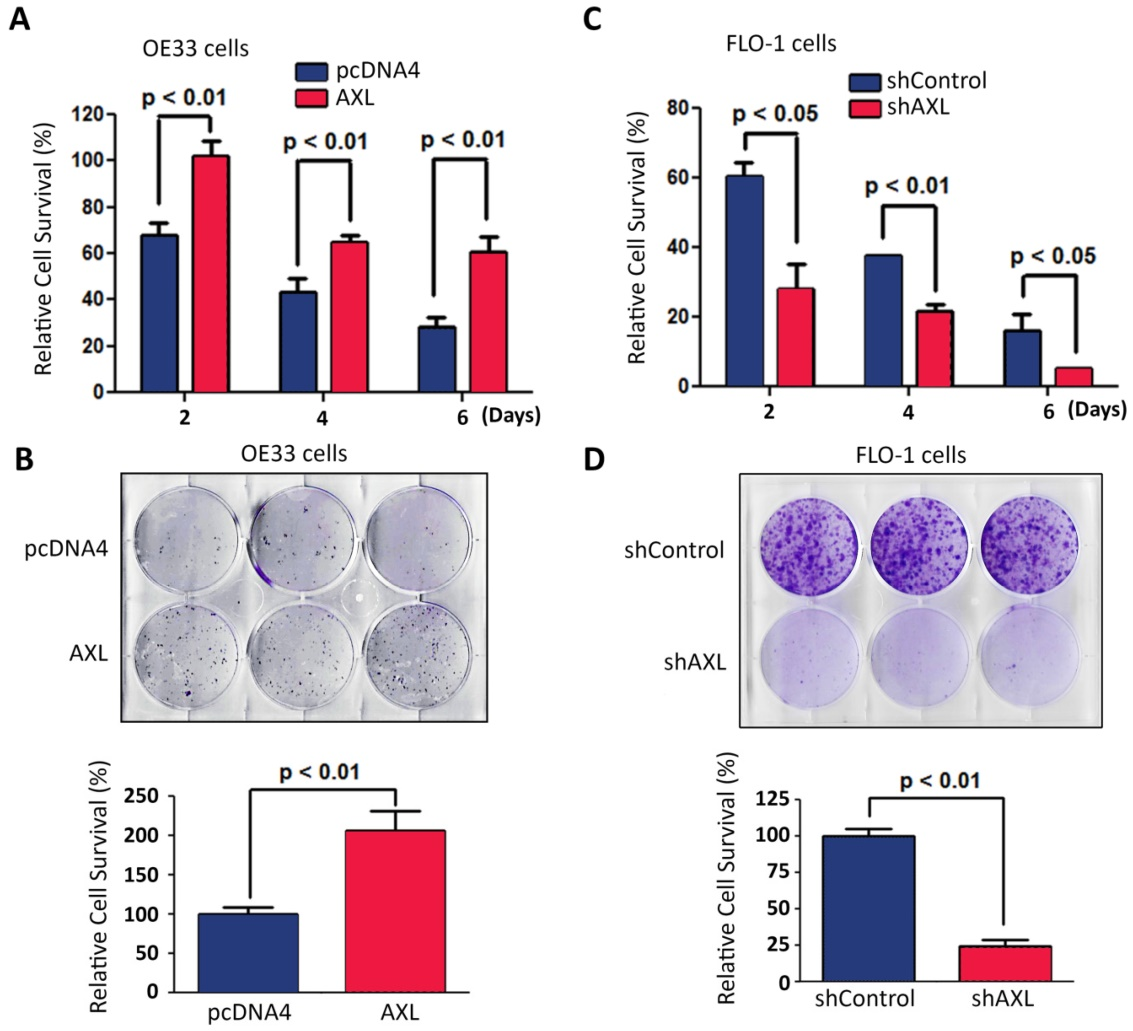
** AXL expression promotes cancer cell survival in response to serum deprivation.** Overexpression of AXL in OE33 cells significantly promoted cell survival in response to serum deprivation for the indicated time points as measured by Trypan blue viability assay (**A**) and clonogenic survival assay (**B**). Knocking down of AXL expression in FLO-1 cells significantly decreased cell survival in response to serum deprivation for the indicated time points as assessed by Trypan blue viability assay (**C**) and clonogenic survival assay (**D**). The data are representative of at least three independent experiments.

We investigated the pro-survival function of AXL in a serum deprivation cell model. This model was used to mimic the limited nutrients availability for solid tumors because of poor vascularization in* vivo*. Overexpression of AXL in OE33 cells significantly enhanced cell survival relative to control cells in response to serum deprivation as demonstrated by Trypan blue cell viability (*p <* 0.01, Figure 4A) and clonogenic survival assays (*p <* 0.01, Figure 4B). In contrast, knocking down of AXL expression in FLO-1 cells significantly decreased cell survival relative to control cells in response to serum deprivation as indicated by Trypan blue cell viability (*p <* 0.05, Figure 4C) and clonogenic survival assays (*p <* 0.01, Figure 4D).

**Figure 5:**
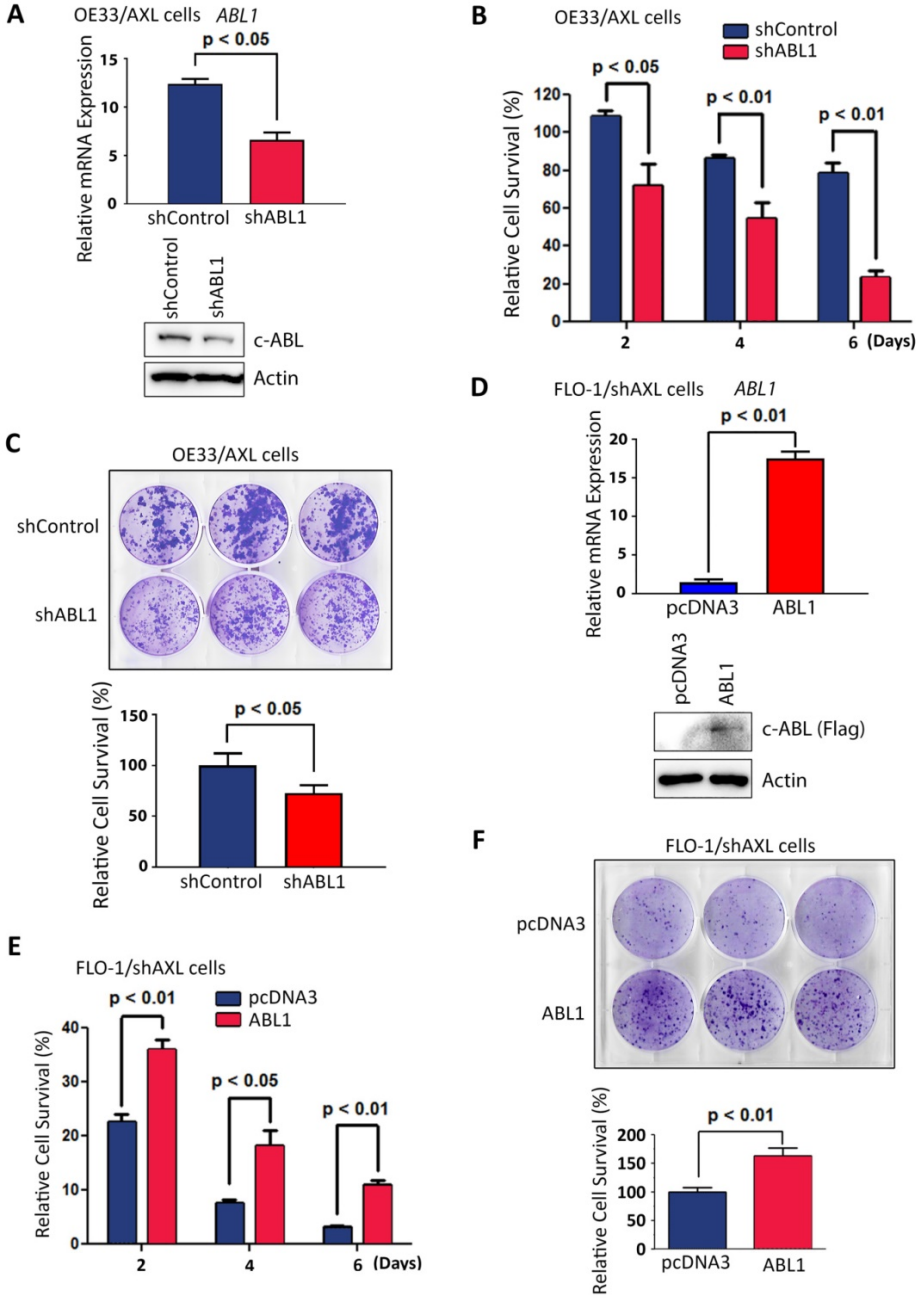
** AXL pro-survival function requires c-ABL expression. A,** OE33/AXL cells were transduced with Control shRNA or a cocktail of five ABL1 shRNA lentivirus particles and c-ABL expression was evaluated by qRT-PCR (upper panel) and Western blotting (lower panel). Knocking down of c-ABL expression in OE33/AXL cells significantly decreased cell survival in response to serum deprivation for the indicated time points as assessed by Trypan blue viability assay (**B**) and clonogenic survival assay (**C**). **D,** FLO-1/shAXL cells were stably transfected with pcDNA3 or ABL1-Flag plasmids and c-ABL expression was assessed by qRT-PCR (upper panel) and Western blot analysis (lower panel). The reconstitution of c-ABL expression in FLO-1/shAXL cells significantly increased cell survival for the indicated time points in response to serum deprivation as evaluated by Trypan blue viability assay (**E**) and clonogenic survival assay (**F**). The data are representative of at least three independent experiments.

Based on our data showing that AXL positively regulates c-ABL protein expression (Figure 3), we investigated whether the pro-survival function of AXL requires c-ABL expression. Therefore, we knocked down the expression of c-ABL in high c-ABL expressing OE33/AXL cells (Figure 5A) and checked if the pro-survival function of AXL could be impaired in these cells. Cell viability and clonogenic survival assays data indicated that knocking down of c-ABL expression in OE33/AXL cells significantly decreased cell survival relative to control cells in response to serum deprivation as shown by Trypan blue survival assay (*p <* 0.05, Figure 5B) and clonogenic survival assay (*p <* 0.05, Figure 5C). Furthermore, we reconstituted the expression of c-ABL in low c-ABL-expressing FLO-1/shAXL cells (Figure 5D) and checked if the pro-survival function of AXL could be rescued in these cells. The reconstitution of c-ABL expression in FLO-1/shAXL cells significantly enhanced survival relative to control cells in response to serum deprivation as shown by cell viability assay (*p <* 0.05, Figure 5E) and clonogenic survival assay (*p <* 0.01, Figure 5F). Together, these data strongly suggested that the pro-survival function of AXL requires c-ABL expression in EAC cells.

### AXL-ABL axis promotes cell survival by mostly counteracting serum deprivation-induced necrosis

**Figure 6:**
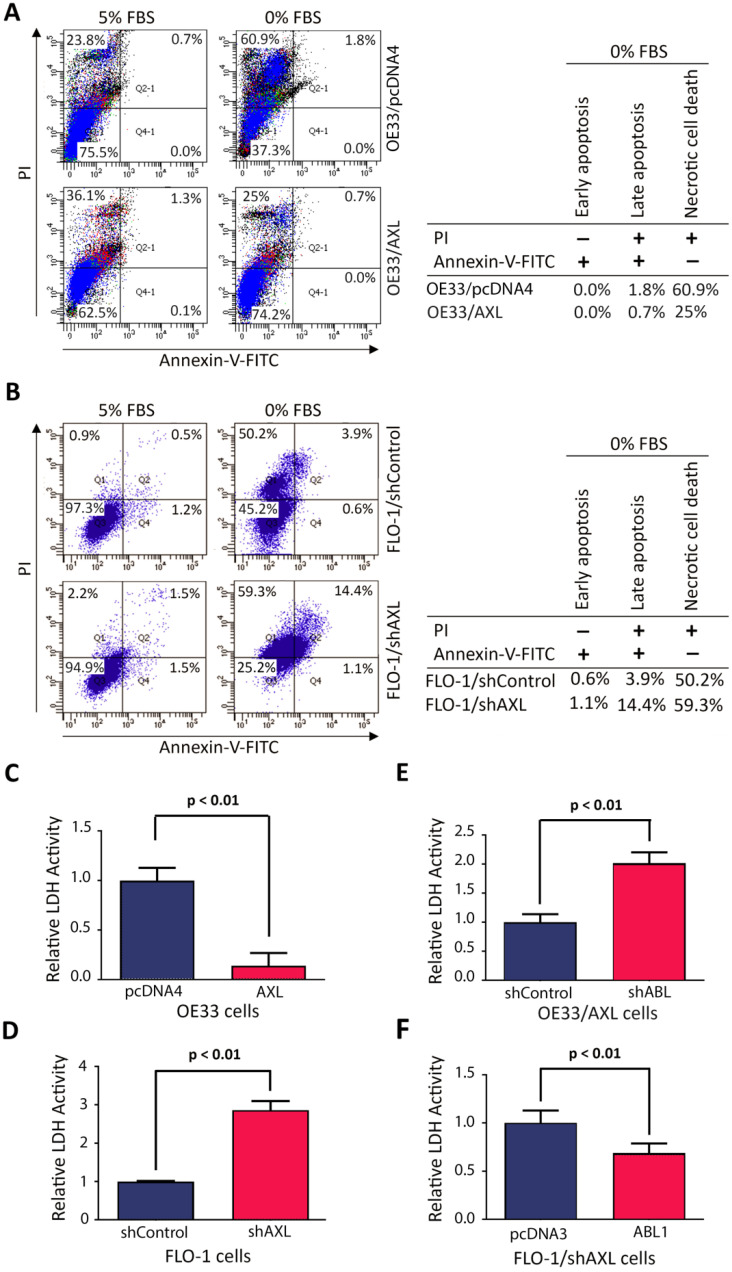
** AXL requires c-ABL expression to counteract mainly serum deprivation-induced necrosis in cancer cells. A,** Overexpression of AXL in OE33 cells decreased necrotic cells death relative to control cells in response to serum deprivation (48 h) as evaluated by Annexin-V/PI staining and FACS analysis. **B,** Knockdown of AXL expression in FLO-1 cells increased apoptosis and necrosis (59.3% vs 50.2%) relative to control cells in response to serum deprivation (24 h). LDH activity assay analysis indicated that overexpression of AXL in OE33 cells significantly decreased necrosis (**C**) and knocking down of AXL in FLO-1 cells significantly increased necrosis (**D**) relative to control cells in response to serum deprivation (24 h). Knocking down of c-ABL expression in OE33/AXL cells significantly increased necrosis (**E**) and the reconstitution of c-ABL expression in FLO-1/shAXL cells significantly reduced necrosis (**F**) relative to control cells relative to control cells in response to serum deprivation (24 h). The data are representative of three independent experiments.

We investigated the cell death type induced by serum deprivation in EAC cells. We cultured cells in media with 5% FBS or 0% FBS for 48 h and the cells were then stained with Annexin-V-FITC and propidium iodide (PI), followed by FACS analysis. The data revealed that serum deprivation induced death in 25.7% of OE33/AXL cells as opposed to 62.7% of OE33/pcDNA4 cells (Figure 6A). In addition, the major type of cell death was necrosis (OE33/AXL, 25%; OE33/pcDNA4, 60.9%) and very little apoptosis (OE33/AXL, 0.7%; OE33/pcDNA4, 1.8%) (Figure 6A). Using another EAC cell model, we found that 24 h-serum deprivation induced death in 74.8% of FLO-1/shAXL cells as opposed to 54.7% of FLO-1/shControl cells (Figure 6B). Further data analysis indicated that the major type of cell death induced by serum deprivation was necrosis (FLO-1/shAXL, 59.3%; FLO-1/shControl, 50.2%) and to a lesser degree was apoptosis (FLO-1/shAXL, 15.5%; FLO-1/shControl, 4.5%) (Figure 6B). These data clearly indicated that AXL promotes cancer cell survival through counteracting serum deprivation-induced necrosis and apoptosis.

As our data revealed that necrosis was the major cell death type induced by serum deprivation, we sought to confirm the pro-survival role of AXL in regulating necrotic cell death using lactate dehydrogenase (LDH) activity assay. LDH is a stable cytoplasmic enzyme and released into the cell culture medium in response to damaged plasma membrane generally caused by necrosis. The data showed that overexpression of AXL in OE33 cells significantly reduced LDH activity as compared to control cells in response to serum deprivation (*p <* 0.01, Figure 6C). Conversely, knocking down of AXL expression in FLO-1 cells significantly increased LDH activity relative to control cells in response to serum deprivation (*p <* 0.01, Figure 6D). We next investigated whether the counteraction of necrosis by AXL required c-ABL expression. The results showed that knocking down of c-ABL expression in OE33/AXL cells significantly increased LDH activity relative to control cells in response to serum deprivation (*p <* 0.01, Figure 6E). Additionally, the reconstitution of c-ABL expression in FLO-1/shAXL cells significantly decreased LDH activity as compared to control cells in response to serum deprivation (*p <* 0.01, Figure 6F). These data confirmed that AXL-dependent reduction of necrotic cell death requires c-ABL expression in a serum deprivation cell model. Together, we demonstrated that AXL-ABL axis promotes cell survival through counteracting mainly serum deprivation-induced necrotic cell death.

## Discussion

Esophageal adenocarcinoma is a highly aggressive malignancy with very poor prognosis because of late diagnosis and limited therapeutic options 5, 23. Enhancing our understanding of the molecular mechanisms that drive the pathogenesis of esophageal adenocarcinoma may lead to identification of novel prognostic biomarkers or therapeutic targets. Except for our previous report on the implication of c-ABL in mediating AXL-dependent cisplatin resistance in EAC 8, no studies have investigated the molecular and functional relationship between these two proteins in solid tumors. In the current study, we sought to further explore the relationship between AXL and c-ABL and evaluate its clinicopathological significance in EAC.

Our IHC data analysis revealed AXL and c-ABL overexpression in 55% and 66% of EAC tissue samples, respectively, and a relatively strong association between AXL and c-ABL protein expression, suggesting a potential molecular and functional relationship. In line with these findings and in support of an important role for AXL in EAC, a previous study indicated that the increasing expression of AXL was associated with Barrett's tumorigenesis 6. The oncogenic form of c-ABL, which is a constitutively active fusion protein (BCR-ABL), has been well established in hematopoietic malignancies such as chronic myeloid leukemia 12. Although very little is known about the expression and function of c-ABL in EAC, several studies reported that the increased protein expression and activation of c-ABL have been associated with pro-tumorigenic functions in solid tumors 13, 15. Some clinicopathological features, including age, tumor stage, and lymph node status in EAC patients were significantly associated with both AXL and c-ABL protein expression, demonstrating an identical clinicopathological association profile. Our investigation of the prognostic value of AXL and c-ABL indicated that high expression levels of these proteins in EAC patients were significantly associated with low patient survival and poor prognosis. Our results support the reports from other studies showing a strong association of AXL expression with poor prognosis in solid malignancies 24. In addition, activation and overexpression of c-ABL have been associated with poor clinical outcome in solid tumors 13, 25.

The co-overexpression and strong association between AXL and c-ABL in EAC cells and tissue samples compelled us to investigate the molecular relationship between these two proteins. Indeed, we found that AXL positively regulates c-ABL protein expression through enhancing cap-dependent translation. Our data indicate that AXL expression mediates phosphorylation of EIF4E, a major regulator of cap-dependent translation, and increases the translated polysomal mRNA level of *ABL1.* We found that AXL-induced phosphorylation of EIF4E is independent of ERK1/2 and P38 MAPK pathways, which are known to regulate EIF4E phosphorylation through Mnk1/2 26, in our EAC cell models (data not shown). It is unlikely that AXL tyrosine kinase is directly implicated in the phosphorylation of EIF4E at S209 residue. Notably, regulation of EIF4E phosphorylation was shown to involve the protein phosphatase 2A (PP2A) in EAC cells 27. Future studies will be required to identify the underlying molecular mechanism in our cell system.

We investigated whether c-ABL mediates the AXL pro-survival function in serum-deprivation cell models. Indeed, we confirmed that AXL promotes cell survival in response to serum deprivation and c-ABL expression is required to mediate the AXL pro-survival function in EAC cells, mainly through counteracting necrotic cell death. These findings further support the pro-tumorigenic function of AXL and c-ABL in EAC. The implication of c-ABL in mediating cell survival has been demonstrated in solid tumor cells. For instance, genetic knockdown or pharmacological inhibition (imatinib; STI571) of c-ABL was reported to sensitize breast cancer cells to lapatinib 18. Similarly, aggressive breast cancer cells have been shown to be dependent on activated c-ABL for proliferation and survival 15. Conversely, c-ABL has been shown to mediate apoptosis in response to DNA damage, and sequestration of c-ABL by AXL in the cytoplasm enhances cancer cell survival in EAC cells 8. This dual function of c-ABL may be dependent on its dysregulated intracellular localization (cytoplasmic *versus* nuclear) and the type of stress cancer cells encounter in the microenvironment. Our IHC data indicated that c-ABL protein expression was mostly cytoplasmic in EAC tissue samples. In line with our observation, high cytoplasmic c-ABL protein expression has been reported in breast and thyroid cancers 13, 28, and was shown to promote EMT and invasive activity in solid tumors 13, 29.

The dependency of the pro-survival function of AXL on c-ABL expression provides an expansion of therapeutic opportunities for EAC patients. Several clinical trials are currently underway in the U.S. to investigate AXL selective small molecule inhibitors such as BGB324/R428 in Melanoma (NCT02872259-Phase I & II) and TP-0903 in solid tumors (NCT03572634-Phase I & II). Unfortunately, there are currently no clinical studies to assess these inhibitors in EAC. Our results suggest that targeting c-ABL with the clinically approved cancer drug imatinib alone or in combination with AXL inhibitors could be an effective therapeutic intervention in EAC.

## Conclusion

**Figure 7:**
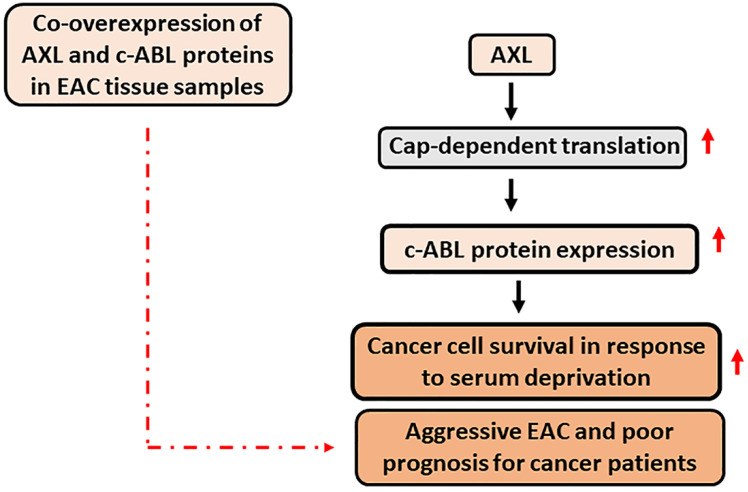
** A schematic representation depicting the role of AXL and c-ABL in EAC.** In summary, AXL upregulates c-ABL protein expression through activation of cap-dependent translation in EAC cells. The AXL pro-survival function depends on c-ABL expression in response to serum deprivation, and co-overexpression of AXL and c-ABL proteins in human EAC samples is significantly associated with aggressive EAC and poor prognosis for cancer patients. Solid black arrow depicts positive regulation. Solid red arrow indicates upregulation of expression or activity. Broken red arrow shows significant statistical association.

This study demonstrated that AXL and c-ABL are co-overexpressed in 49% of EAC samples and established that the expression of these proteins is associated with aggressive disease and poor prognosis. The data indicated that the pro-survival function of AXL depends on c-ABL expression in response to serum deprivation. In addition, AXL promotes c-ABL protein expression through upregulation of cap-dependent translation. A summary of the data is shown in a schematic diagram (Figure 7). Our novel findings highlight the importance of AXL and c-ABL co-overexpression as a valuable prognostic biomarker and targeting these proteins could be an effective therapeutic approach in EAC or possibly other types of solid tumors.

## Supplementary Material

Supplementary figures and tables.

## Figures and Tables

**Table 1:** Association between AXL expression and clinicopathological features

	N	Low (N = 18)	Medium (N = 6)	High (N = 29)	*P* value
**Gender**	53				0.905^a^
Female		11% (2)	17% (1)	10% (3)	
Male		89% (16)	83% (5)	90% (26)	
**Age**	53	48.25, **54.00**, 56.00	68.50, **70.50,** 71.00	62.00, **65.00,** 71.00	<0.001^b^
***Age categories***	53				<0.001^c^
<60		94% (17)	0% (0)	7% (2)	
≥60		6% (1)	100% (6)	93% (27)	
**Tumor size (cm)**	52	1.00, **1.50,** 4.00	1.25, **2.52,** 3.75	1.00, **3.00,** 5.25	0.799^b^
***Tumor categories***	52				0.35^a^
<3 cm		61% (11)	50% (3)	39% (11)	
≥3 cm		39% (7)	50% (3)	61% (17)	
**Lymph nodes**	53				<0.001^c^
Negative		100% (18)	0% (0)	7% (2)	
Positive		0% (0)	100% (6)	93% (27)	
***Stage***	53				0.001^a^
0		22% (4)	17% (1)	0% (0)	
1		56% (10)	33% (2)	14% (4)	
2		17% (3)	0% (0)	59% (17)	
3		6% (1)	50% (3)	24% (7)	
4		0% (0)	0% (0)	3% (1)	
**N Classification**	53				0.31^a^
N0		39% (7)	67% (4)	52% (15)	
N1		33% (6)	17% (1)	10% (3)	
N2		28% (5)	17% (1)	38% (11)	
**T Classification**	53				0.634^a^
T1		50% (9)	67% (4)	48% (14)	
T2		17% (3)	17% (1)	34% (10)	
T3		28% (5)	17% (1)	10% (3)	
T4		6% (1)	0% (0)	7% (2)	
**Differentiation**	49				0.507^a^
Intestinal		12% (2)	17% (1)	0% (0)	
Moderately diff.		59% (10)	33% (2)	46% (12)	
Mod-poorly diff.		12% (2)	17% (1)	23% (6)	
Poorly diff.		18% (3)	17% (1)	19% (5)	
Well differentiated		0% (0)	17% (1)	12% (3)	

N, number of EAC samples. Numbers after proportions are frequencies. Tests used: a, Pearson test; b, Kruskal-Wallis test; c, Fisher's exact test.

**Table 2:** Association between c-ABL expression and clinicopathological features

	N	Low (N = 7)	Medium (N = 11)	High (N = 35)	*P* value
**Gender**	53				0.657^a^
Female		14% (1)	18% (2)	9% (3)	
Male		86% (6)	82% (9)	91% (32)	
**Age**	53	51.5, **56.0,** 57.0	50.5, **55.0,** 58.0	62.0, **68.0,** 71.0	<0.001^b^
***Age categories***	53				<0.001^c^
<60		100% (7)	73% (8)	11% (4)	
≥60		0% (0)	27% (3)	89% (31)	
**Tumor size (cm)**	52	1.00, **3.00,** 4.00	1.00, **1.00,** 4.00	1.00, **3.00,** 4.75	0.925^b^
***Tumor categories***	52				0.507^a^
<3 cm		43% (3)	64% (7)	44% (15)	
≥3 cm		57% (4)	36% (3)	56% (19)	
**Lymph nodes**	53				<0.001^c^
Negative		100% (7)	82% (9)	11% (4)	
Positive		0% (0)	18% (2)	89% (31)	
Stage	53				0.017^a^
0		29% (2)	18% (2)	3% (1)	
1		57% (4)	55% (6)	17% (6)	
2		14% (1)	9% (1)	51% (18)	
3		0% (0)	18% (2)	26% (9)	
4		0% (0)	0% (0)	3% (1)	
**N Classification**	53				0.319^a^
N0		57% (4)	36% (4)	51% (18)	
N1		29% (2)	36% (4)	11% (4)	
N2		14% (1)	27% (3)	37% (13)	
**T Classification**	53				0.212^a^
T1		43% (3)	64% (7)	49% (17)	
T2		0% (0)	18% (2)	34% (12)	
T3		43% (3)	18% (2)	11% (4)	
T4		14% (1)	0% (0)	6% (2)	
**Differentiation**	49				0.065^a^
Intestinal		33% (2)	9% (1)	0% (0)	
Moderately diff.		33% (2)	73% (8)	44% (14)	
Mod-poorly diff.		17% (1)	9% (1)	22% (7)	
Poorly diff.		17% (1)	9% (1)	22% (7)	
Well differentiated		0% (0)	0% (0)	12% (4)	

N, number of EAC samples. Numbers after proportions are frequencies. Tests used: a, Pearson test; b, Kruskal-Wallis test; c, Fisher's exact test.

## Abbreviations

CHX: Cycloheximide
EAC: Esophageal adenocarcinoma
EGFR: Epidermal growth factor receptor
EIF4E: Eukaryotic translation initiation factor
HER2: Human epidermal growth factor receptor 2
HR: Hazard ratio
LDH: Lactate dehydrogenase
IHC: Immunohistochemical
qRT-PCR: Quantitative real-time PCR
TMA: Tissue microarray
TrIP-Chip: Translational chromatin immunoprecipitation
TRAIL: Tumor necrosis factor-related apoptosis-inducing ligand

## References

[1] Edgren G, Adami HO, Weiderpass E, Nyren O (2013). A global assessment of the oesophageal adenocarcinoma epidemic. *Gut*.

[2] Lagergren J, Lagergren P (2013). Recent developments in esophageal adenocarcinoma. *CA Cancer J Clin*.

[3] Cook MB, Corley DA, Murray LJ, Liao LM, Kamangar F, Ye W (2014). Gastroesophageal reflux in relation to adenocarcinomas of the esophagus: a pooled analysis from the Barrett's and Esophageal Adenocarcinoma Consortium (BEACON). *PLoS One*.

[4] Anaparthy R, Sharma P (2014). Progression of Barrett oesophagus: role of endoscopic and histological predictors. *Nat Rev Gastroenterol Hepatol*.

[5] Coleman HG, Xie SH, Lagergren J (2018). The Epidemiology of Esophageal Adenocarcinoma. *Gastroenterology*.

[6] Hector A, Montgomery EA, Karikari C, Canto M, Dunbar KB, Wang JS (2010). The Axl receptor tyrosine kinase is an adverse prognostic factor and a therapeutic target in esophageal adenocarcinoma. *Cancer Biol Ther*.

[7] Hong J, Belkhiri A (2013). AXL mediates TRAIL resistance in esophageal adenocarcinoma. *Neoplasia*.

[8] Hong J, Peng D, Chen Z, Sehdev V, Belkhiri A (2013). ABL regulation by AXL promotes cisplatin resistance in esophageal cancer. *Cancer Res*.

[9] Hong J, Maacha S, Belkhiri A (2018). Transcriptional upregulation of c-MYC by AXL confers epirubicin resistance in esophageal adenocarcinoma. *Mol Oncol*.

[10] Shaul Y, Ben-Yehoyada M (2005). Role of c-Abl in the DNA damage stress response. *Cell Res*.

[11] Greuber EK, Smith-Pearson P, Wang J, Pendergast AM (2013). Role of ABL family kinases in cancer: from leukaemia to solid tumours. *Nat Rev Cancer*.

[12] Hantschel O, Superti-Furga G (2004). Regulation of the c-Abl and Bcr-Abl tyrosine kinases. *Nat Rev Mol Cell Biol*.

[13] Srinivasan D, Plattner R (2006). Activation of Abl tyrosine kinases promotes invasion of aggressive breast cancer cells. *Cancer Res*.

[14] Rikova K, Guo A, Zeng Q, Possemato A, Yu J, Haack H (2007). Global survey of phosphotyrosine signaling identifies oncogenic kinases in lung cancer. *Cell*.

[15] Srinivasan D, Sims JT, Plattner R (2008). Aggressive breast cancer cells are dependent on activated Abl kinases for proliferation, anchorage-independent growth and survival. *Oncogene*.

[16] Birge RB, Fajardo JE, Mayer BJ, Hanafusa H (1992). Tyrosine-phosphorylated epidermal growth factor receptor and cellular p130 provide high affinity binding substrates to analyze Crk-phosphotyrosine-dependent interactions in vitro. *J Biol Chem*.

[17] Tanos B, Pendergast AM (2006). Abl tyrosine kinase regulates endocytosis of the epidermal growth factor receptor. *J Biol Chem*.

[18] Lo YH, Ho PC, Zhao H, Wang SC (2011). Inhibition of c-ABL sensitizes breast cancer cells to the dual ErbB receptor tyrosine kinase inhibitor lapatinib (GW572016). *Anticancer Res*.

[19] Belkhiri A, Zaika A, Pidkovka N, Knuutila S, Moskaluk C, El-Rifai W (2005). Darpp-32: a novel antiapoptotic gene in upper gastrointestinal carcinomas. *Cancer Res*.

[20] Dematteo RP, Heinrich MC, El-Rifai WM, Demetri G (2002). Clinical management of gastrointestinal stromal tumors: before and after STI-571. *Hum Pathol*.

[21] Kudo K, Xi Y, Wang Y, Song B, Chu E, Ju J (2010). Translational control analysis by translationally active RNA capture/microarray analysis (TrIP-Chip). *Nucleic Acids Res*.

[22] Maacha S, Hong J, von Lersner A, Zijlstra A, Belkhiri A (2018). AXL Mediates Esophageal Adenocarcinoma Cell Invasion through Regulation of Extracellular Acidification and Lysosome Trafficking. *Neoplasia*.

[23] Ku GY, Ilson DH (2009). Preoperative therapy for esophageal cancer. *Gastroenterol Clin North Am*.

[24] Zhang S, Xu XS, Yang JX, Guo JH, Chao TF, Tong Y (2018). The prognostic role of Gas6/Axl axis in solid malignancies: a meta-analysis and literature review. *Onco Targets Ther*.

[25] Zhou S, Tang L, Wang H, Dai J, Zhang J, Shen L (2013). Overexpression of c-Abl predicts unfavorable outcome in epithelial ovarian cancer. *Gynecol Oncol*.

[26] Hay N (2010). Mnk earmarks eIF4E for cancer therapy. *Proc Natl Acad Sci U S A*.

[27] Katsha A, Wang L, Arras J, Omar OM, Ecsedy J, Belkhiri A (2017). Activation of EIF4E by Aurora Kinase A Depicts a Novel Druggable Axis in Everolimus-Resistant Cancer Cells. *Clin Cancer Res*.

[28] Podtcheko A, Ohtsuru A, Tsuda S, Namba H, Saenko V, Nakashima M (2003). The selective tyrosine kinase inhibitor, STI571, inhibits growth of anaplastic thyroid cancer cells. *J Clin Endocrinol Metab*.

[29] Yang L, Lin C, Liu ZR (2006). P68 RNA helicase mediates PDGF-induced epithelial mesenchymal transition by displacing Axin from beta-catenin. *Cell*.

