# Supplementary material for: Co-overexpression of AXL and c-ABL predicts a poor prognosis in esophageal adenocarcinoma and promotes cancer cell survival
Source: J Cancer. 2020 Aug 8;11(20):5867–79. doi: 10.7150/jca.47318 (PMC7477426; doi:10.7150/jca.47318)
Supplement: Supplementary file 1 — Supplementary figures and tables. [file jcav11p5867s1.pdf]

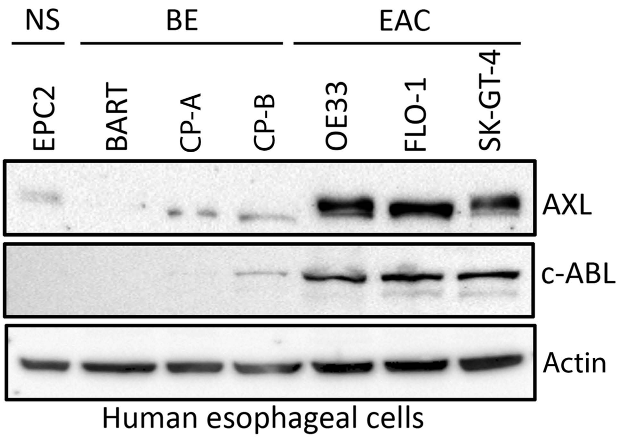

Supplemental Figure S1. Co-overexpression of AXL and c-ABL proteins in esophageal adenocarcinoma cell lines. Proteins from normal esophageal squamous epithelial cells (NS; EPC2), Barrett's cells (BE; BART, CP-A, and CP-B), and esophageal adenocarcinoma cells (EAC; OE33, FLO-1, and SK-GT-4) were subjected to Western blot analysis of AXL and c-ABL proteins.

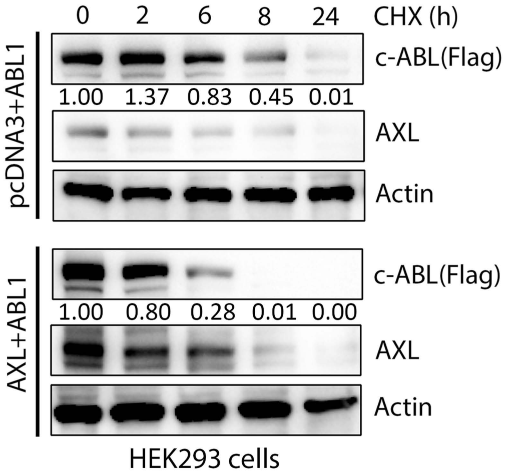

S**upplemental Figure S2. AXL expression does not enhance c-ABL protein stability.** HEK293 cells were transiently co-transfected with ABL1 and pcDNA3 or AXL and treated with CHX for the indicated timepoints. Western blot analysis was used to assess c-ABL protein levels. The data showed that c-ABL protein stability was not increased by AXL expression.

Supplemental Table S1 . Co-overexpression of AXL and c-ABL proteins in esophageal adenocarcinomas

	N	EAC $N = 53$		Normal $N = 11$		p-value
AXL index score	64					$<0.001^2$
0		13%	(7)	0%	(0)	
1		21%	(11)	64%	(7)	
2		11%	(6)	36%	(4)	
3		55%	(29)	0%	(0)	
c-ABL index score	64					$<0.001^2$
0		6%	(3)	45%	(5)	
2		8%	(4)	27%	(3)	
4		21%	(11)	27%	(3)	
8		26%	(14)	0%	(0)	
12		40%	(21)	0%	(0)	

N, the number of samples and numbers after proportions are frequencies. AXL (55%, IHC index score = 3) and c-ABL (66%, IHC index score ≥ 8) are frequently overexpressed in esophageal adenocarcinomas. 2, Pearson test was used to assess the differences in AXL and c-ABL index scores between normal and tumor tissue samples.

Supplemental Table S2: descriptive patient characteristics

	N	
Gender	53	
Female		11% (6)
Male		89% (47)
Age	53	56 63 70
Age categories	53	*a b c*
<60		36% (19)
≥60		64% (34)
Race/Ethnicity	44	
Latino		11% (5)
White		89% (39)
AXL index score	53	
0		13% (7)
1		21% (11)
2		11% (6)
3		55% (29)
AXL	53	
<2		34% (18)
≥2		66% (35)
c-ABL index score	53	
0		6% (3)
2		8% (4)
4		21% (11)
8		26% (14)
12		40% (21)
c-ABL	53	
<8		34% (18)
≥8		66% (35)
Location	53	
distal esophagus		4% (2)
Esophagus		51% (27)
G-E Junction		45% (24)
Tumor size (cm)	52	1.0 2.5 4.0
Tumor categories	52	
<3cm		48% (25)
≥3cm		52% (27)
Barrett's	27	
Yes		93% (25)
No		7% (2)
Surgery performed	49	
Yes		22% (11)
No		78% (38)

N, number of patients. Numbers after proportions are frequencies. *a b c* represent the lower quartile *a*, the median *b*, and the upper quartile *c* for continuos variables.

Supplemental Table S2: ***(continued)***

	N		
Tumor core histology	51		
Diffuse		8%	(4)
Intestinal		73%	(37)
mixed		4%	(2)
Papillary		2%	(1)
poorly diff		4%	(2)
Signet-Ring		8%	(4)
well diff		2%	(1)
Differentiation	49		
intestinal		6%	(3)
Moderately-differentiated		49%	(24)
Mod-poorly differentiated		18%	(9)
Poorly differentiated		18%	(9)
Well-differentiated		8%	(4)
Lymph Nodes	53		
Negative		38%	(20)
Positive		62%	(33)
Stage	53		
0		9%	(5)
1		30%	(16)
2		38%	(20)
3		21%	(11)
4		2%	(1)
N Classification	53		
N0		49%	(26)
N1		19%	(10)
N2		32%	(17)
T Classification	53		
T1		51%	(27)
T2		26%	(14)
T3		17%	(9)
T4		6%	(3)
Number Positive Nodes	51		
0		47%	(24)
1		24%	(12)
2		10%	(5)
3		6%	(3)
4		4%	(2)
5		2%	(1)
6		4%	(2)
7		2%	(1)

N, number of patients. Numbers after proportions are frequencies. *a b c* represent the lower quartile *a,* the median *b,* and the upper quartile *c* for continuos variables.

Supplemental Table S2: ***(continued)***

	N	
8		2% (1)
Number Total Nodes	50	5 8 14
Cancer reccurence	53	*a b c*
Yes		96% (51)
No		4% (2)
Recurrence after chemo	53	
Yes		100% (53)
No		0% (0)
Recurrence Free Survival	53	114 845 1890
location.of.tumor.recurrence	27	*a b c*
anastamosis		4% (1)
Bone		4% (1)
Brain		7% (2)
Esophagus		4% (1)
Femur		4% (1)
Liver		11% (3)
Lung		7% (2)
Lymph node (supraclavicular)		4% (1)
Mediastinum		7% (2)
NA		19% (5)
Neck		4% (1)
Perirenal		4% (1)
Pleura		15% (4)
Ribs		4% (1)
Stomach		4% (1)
Died of cancer	53	
Yes		100% (53)
No		0% (0)
Follow-up period	53	45 450 1740
Recurrence survival days	53	114 845 1890
Post Op CT	34	*a b c*
Yes		41% (14)
No		59% (20)

N, number of patients. Numbers after proportions are frequencies. *a b c* represent the lower quartile *a,* the median *b,* and the upper quartile *c* for continuos variables.

Supplemental Table S3. List of quantitative real-time PCR primers sequences

HPRT1 (forward)	5'-ACCCTTTCCAAATCCTCAGC-3'
HPRT1 (reverse)	5'-GTTATGGCGACCCGCAG-3'
AXL (forward)	5'-GAAGGTACCATGACAACCCAGGCAAAGTG-3'
AXL (reverse)	5'-GAACTCGAGACGCCATGGGTGCCAAAC-3'
ABL1 (forward)	5'-AAGCCGCTCGTTGGAACTC-3'
ABL1 (reverse)	5'-AGACCCGGAGCTTTTCACCT-3'
EIF4E1 (forward)	5'-GAAACCACCCCTACTCCTAATCC-3'
EIF4E1 (reverse)	5'-AGAGTGCCCATCTGTTCTGTA-3'
EIF4E2 (forward)	5'- ACAACAAGTTCGACGCTTTGA-3'
EIF4E2 (reverse)	5'- TCTCTTGCTACTGCTCTGATTCT-3'
EIF4E3 (forward)	5'- GAGTTAGTGTCAGTGTTCGGG-3'
EIF4E3 (reverse)	5'- TGTGGGGCAGAAGTTCATAGA-3'

Cell culture and reagents

The human EAC cancer cell line, SK-GT-4, was purchased from Sigma-Aldrich (St. Louis, MO, USA). SK-GT-4 cells were cultured in Dulbecco's modified Eagle's medium (DMEM; GIBCO, Carlsbad, CA, USA) supplemented with 5% fetal bovine serum (FBS, Invitrogen Life technologies, Carlsbad, CA, USA) and 1% penicillin/streptomycin (GIBCO). CP-A and CP-B cells were obtained from ATCC (Manassas, VA, USA) and BAR-T cells [1] were cultured in DMEM/F12 medium supplemented with 0.4 µg/ml hydrocortisone, 20 ng/ml recombinant human epidermal growth factor, 20 mg/ml adenine, 140 µg/ml bovine pituitary extract, 0.1% ITS Supplement (Sigma-Aldrich; I1884), 4 mM glutamine, and 5% FBS. EPC2 cells were kindly provided by Dr. Hiroshi Nakagawa (University of Pennsylvania, Philadelphia, PA, USA) and cultured in keratinocyte serum-free medium supplemented with 40 mg/ml bovine pituitary extract and 1 ng/ml EGF (Invitrogen). HEK-293 were obtained from ATCC and maintained in F12 (HAM) medium (GIBCO) supplemented with 10% FBS and 1% penicillin/streptomycin. All cell lines were authenticated using short tandem repeat (STR) profiling (Genetica DNA Laboratories, Burlington, NC, USA). Cycloheximide was purchased from Sigma-Aldrich.

c-ABL protein stability assay

HEK-293 cells were transiently co-transfected with Flag-ABL1 in combination with AXL-Myc-His or pcDNA3 plasmids and cultured for 24 h. The cells were then treated with 80 µg/ml of cycloheximide (CHX) and harvested at 0-, 2-, 6-, 8-, and 24-hour time points. Proteins from each time point were prepared and analyzed by Western blotting to evaluate c-ABL protein

stability. The mean intensity of protein bands was analyzed by densitometry using IMAGEJ (NIH Image) and normalized to β-actin.

References

1 Jaiswal KR, Morales CP, Feagins LA, Gandia KG, Zhang X, Zhang HY *et al*. Characterization of telomerase-immortalized, non-neoplastic, human Barrett's cell line (BAR-T). *Dis Esophagus* 2007; 20: 256-264.
